# Relationship between endometrial VFI values detected by three-dimensional power Doppler ultrasound and pregnancy outcomes in FET patients and prediction of the optimal VFI range—a retrospective cohort study

**DOI:** 10.3389/fmed.2026.1786174

**Published:** 2026-06-05

**Authors:** Run Zhao, Linlin Che, Xiaofang Li, Qianyan Song, Lihong Zhou, Siyu Long, Yu Zhang

**Affiliations:** Affiliated Hospital of Southwest Medical University, Luzhou, Sichuan, China

**Keywords:** frozen–thawed embryo transfer, pregnancy outcome, restricted cubic spline curve, three-dimensional energy Doppler ultrasound examination, VFI value of endometrium

## Abstract

**Background:**

The relationship between the vascular flow index (VFI) of the endometrium and the pregnancy outcome in the frozen embryo transfer (FET) cycle remains controversial, and there is currently no recommended optimal range for VFI.

**Objective:**

This study aimed to explore the relationship between three-dimensional energy Doppler ultrasound-detected VFI and pregnancy outcomes in FET patients and to determine the appropriate range of endometrial VFI.

**Methods:**

A retrospective analysis was conducted on the clinical data of 338 patients who received FET-assisted pregnancy treatment in the Reproductive Medicine Center of our hospital from January 2019 to January 2025. The patients were divided into four groups based on the quartiles of endometrial VFI values. The general data, three-dimensional energy Doppler ultrasound detection indicators, and pregnancy outcomes of the four groups were compared.

**Results:**

The abortion rates in Group 3 and Group 4 were significantly lower than those in Group 1 (*p* < 0.05). The multivariate logistic regression analysis showed that, compared with Group 1, the adjusted odds ratio (OR) values for Group 3 and Group 4 were 0.210 (95% CI 0.055–0.805) and 0.227 (95% CI 0.059–0.870), respectively, suggesting that the risk of abortion in the groups with high endometrial VFI values was significantly reduced (*p* < 0.05). The restricted cubic spline (RCS) analysis indicated a significant linear negative correlation between the endometrial VFI value and the abortion rate (*p* < 0.05), with a threshold of 0.1698045. This finding suggests that, after exceeding this threshold, the abortion rate decreases as the endometrial VFI value increases.

**Conclusion:**

Three-dimensional energy Doppler ultrasound measurement of endometrial VFI values can be used as a predictive indicator for the risk of miscarriage in patients undergoing FET. When the VFI value is greater than 0.17, the pregnancy prognosis of the patients is better. During FET, clinicians can take a VFI of > 0.17 as an important reference index for evaluating endometrial receptivity and determining whether to perform embryo transfer.

## 1 Introduction

With the development of assisted reproductive technology (ART), the frozen–thawed embryo transfer (FET) technique has gradually become an important component of ART due to its advantages, such as increasing the cumulative pregnancy rate, reducing the impact of high estrogen levels in fresh embryo transfer on endometrial receptivity, and preventing the occurrence of ovarian hyperstimulation syndrome (OHSS) (1, 2). However, the pregnancy outcome of FET is influenced by multiple factors. Endometrial receptivity is an important factor affecting embryo implantation and development (3, 4). Therefore, accurately assessing endometrial receptivity and choosing the optimal timing of embryo transfer are of great significance for improving the success rate of FET (5). Endometrial ultrasound examination is a commonly used non-invasive method for evaluating endometrial receptivity. Good endometrial blood perfusion is a necessary condition for embryo implantation and development. A Doppler study of uterine arteries cannot reflect the actual blood flow of the endometrium, while three-dimensional energy Doppler ultrasound can more objectively and reliably measure endometrial and subendometrial blood flow. Among its parameters, the endometrial VFI value, which acts as an indicator reflecting the degree of blood flow abundance, has been gradually applied in the assessment of endometrial receptivity (6, 7). Studies have found that insufficient endometrial blood perfusion may lead to embryo implantation failure or early miscarriage (8, 9); however, the conclusion regarding the relationship between the VFI value of the endometrium and the pregnancy outcomes in FET patients remains inconsistent (10). This study aims to compare the pregnancy outcomes in FET patients with different VFI values to explore the relationship between endometrial VFI values and pregnancy outcomes. At the same time, it uses the RCS analysis to analyze the dose–response relationship between endometrial VFI values and pregnancy outcomes and to predict the appropriate range of endometrial VFI values, thereby providing a reference for clinical practice.

## 2 Materials and methods

### 2.1 Participants

**Figure 1:**
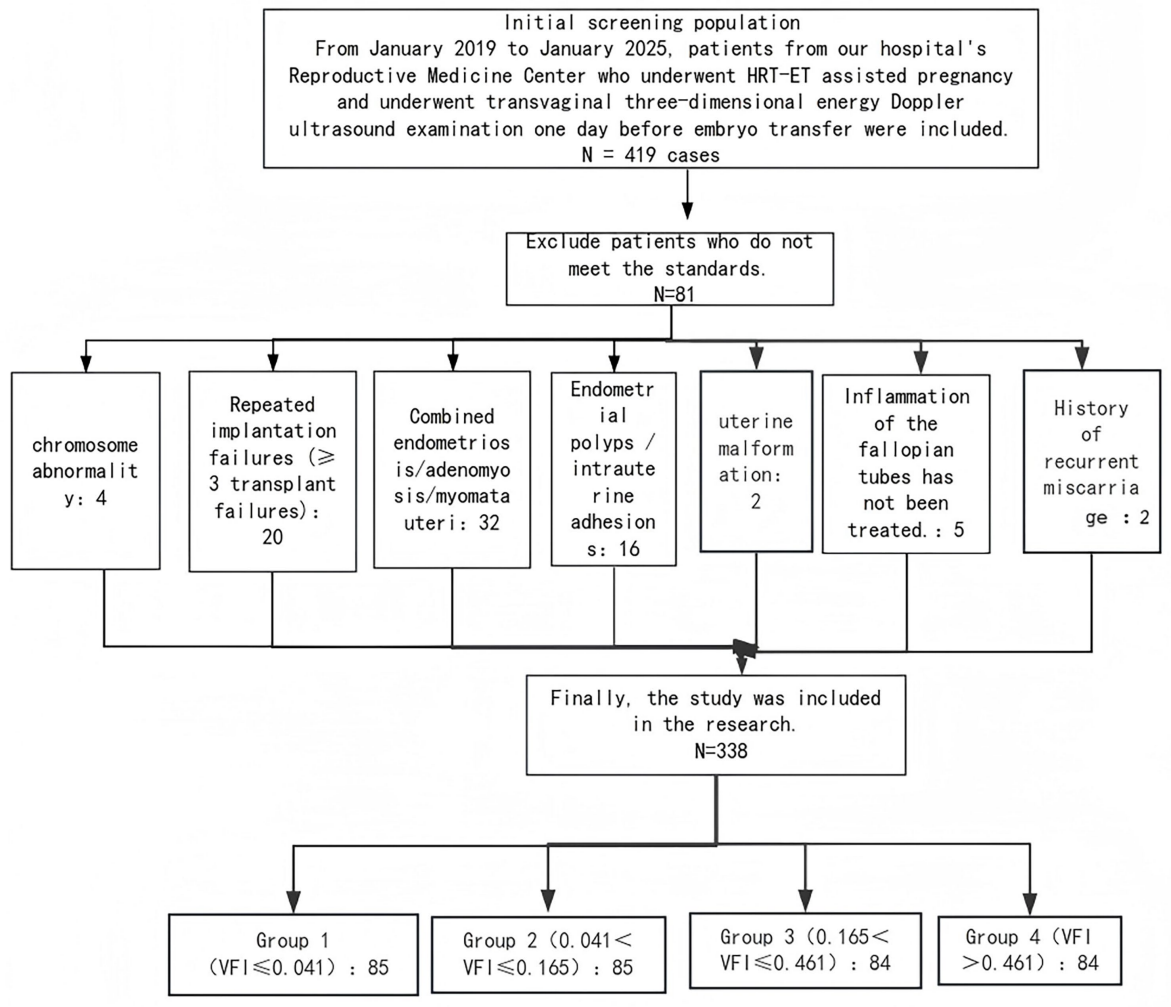
Patient screening flowchart.

This study was conducted in August 2025. A total of 338 patients who underwent FET-assisted pregnancy treatment at the Reproductive Medicine Center of our hospital from January 2019 to January 2025 were included; each patient was included only in the first hormone replacement therapy–frozen embryo transfer (HRT-FET) cycle to avoid data duplication. The patients were divided into four groups based on the quartiles of endometrial VFI values: Group 1 had 85 cases (VFI ≤ 0.041), Group 2 had 85 cases (0.041 < VFI ≤ 0.165), Group 3 had 84 cases (0.165 < VFI ≤ 0.461), and Group 4 had 84 cases (VFI > 0.461). The inclusion criteria were as follows: (1) patients who received FET-assisted pregnancy treatment; (2) those who underwent endometrial preparation using a hormone replacement therapy (HRT) protocol; (3) female patients aged ≤ 40 years; (4) those without severe organic diseases, such as heart, liver, or kidney diseases; (5) those without autoimmune diseases; and (6) male partners with normal semen parameters. The exclusion criteria were as follows: (1) patients with any chromosomal abnormality in either the husband or wife; (2) those with repeated implantation failure (≥ 3 unsuccessful embryo transplants); (3) patients with endometriosis, adenomyosis, or uterine fibroids; (4) those with uterine endometrial polyps and intrauterine adhesions; (5) those with uterine malformations; (6) those with unresolved tubal hydrosalpinx; and (7) those with a history of recurrent miscarriage. The patient screening process is shown in Figure 1. This study was approved by the ethics committee of our hospital (KY2024292).

### 2.2 Research methods

#### 2.2.1 Three-dimensional energy Doppler ultrasound detection method and indicators

Patients included in this study underwent transvaginal three-dimensional energy Doppler ultrasound examination using the Voluson E10 on the day before embryo transfer to evaluate uterine artery blood flow, including endometrial volume (VI), bilateral uterine artery blood flow parameters [pulsatility index (PI) and resistance index (RI)], vascularization index (VI), flow index (FI), and vascularization flow index (VFI) (11). All parameters were independently measured by two senior physicians with extensive experience in reproductive ultrasound. The examination followed a unified standardized operation protocol. The ultrasound examination parameters were set as follows: gain (Gn) was 1.4, frequency (Frq) was set to a medium level, wall filtering (WMF) was adjusted to level 1 (low), pulse repetition frequency (PRF) was 0.9 kHz, and the probe angle was 60°. All indicators were measured repeatedly for ≥ 3 times, and the average value was taken as the final result to reduce the differences between the equipment and the operators and to ensure the stability and repeatability of the data.

#### 2.2.2 Endometrial preparation plan

Hormone replacement therapy (HRT) cycle plan: vaginal ultrasonography was conducted on the second day to fourth day of the menstrual cycle, and estradiol valerate tablets were taken orally (Benjale, Bayer, Germany) at a dosage of 3 mg/time, twice daily. Vaginal ultrasonography and serum E2 and P levels were assessed 14 days after administration. If the endometrium was type A, had a thickness of ≥ 8 mm, and an E2 level of ≥ 150–200 pg./mL, the endometrial preparation was performed with luteal support. The luteal support consisted of oral dydrogesterone tablets (Dufton, Abbott, Netherlands) at a dosage of 10 mg tid, an intramuscular progesterone injection (Zhejiang Xianju, China) at a dosage of 60 mg qd, or oral dydrogesterone tablets at a dosage of 10 mg tid and vaginal progesterone sustained-release gel (Schnaughton, Merck Serono Co., Ltd., Germany) at a dosage of 90 mg qd. The transformation lasted for 4 days, and the recovery and transplantation of the cleavage embryo were conducted on the fifth day. For blastocyst transfer, the transformation lasted for 6 days, and embryo transfer was conducted on the seventh day (12). If the serum E2 level fails to reach the aforementioned level or if the thickness of the endometrium increases by less than 8 mm, the patient should continue to take oral estradiol valerate tablets at a dosage of 3 mg/time twice daily and should undergo vaginal ultrasound and E2 and P tests every 3 days. The cycle will be canceled if the serum E2 level fails to reach the aforementioned level, or if the endometrial thickness remains < 8 mm or ≥16 mm after 17–21 days of taking estradiol valerate tablets.

#### 2.2.3 Fetal cryopreservation and transplantation

The embryos were cryopreserved using the vitrification technique. After the endometrium was prepared to meet the required criteria, the frozen embryos were thawed and their survival status and quality were evaluated. The scoring criteria for cleavage-stage embryos were based on the “Assisted Reproduction Laboratory Techniques” edited by Huang Guoning, the Society for Assisted Reproductive Technology (SART 2010), Alpha Executive, and the Embryology Working Group of ESHRE (2011), regarding the scoring methods for the cleavage-stage embryos. High-quality embryos referred to grade I and II cleavage-stage embryos with a 2PN origin and 7–9 cells on the third day; the scoring for blastocysts primarily used the Gardner scoring system (13), and high-quality embryos referred to blastocysts of grade 4BB or above. After thawing, embryo transplantation was performed under routine ultrasound guidance within 2–4 h. The number of embryos to be transplanted was determined to be 1–2 based on the patient’s age, presence of a scarred uterus, the patient’s personal preference, and previous assisted reproductive history.

#### 2.2.4 Criteria for adjudicating pregnancy outcome

Human chorionic gonadotropin (HCG) positive: 2 weeks after transplantation, serum human chorionic gonadotropin (*β*-hCG) was detected; β-hCG was > 5 IU/L, and the HCG-positive rate was calculated as the number of HCG-positive patients/the number of patients in the transplant cycle ×100%. Clinical pregnancy: The pregnancy sac (intrauterine or extrauterine) was detected using vaginal ultrasound at 4–5 weeks after transplantation. The clinical pregnancy rate is calculated as the number of patients with clinical pregnancy/the number of patients in the transplant cycle×100%. Early abortion is defined as the termination of pregnancy within 12 weeks of gestation when the embryo or fetus is not still viable. The early abortion rate was calculated as the number of spontaneous abortion cycles/the number of clinical pregnancy cycles ×100%.

### 2.3 Statistical analysis

Data analysis was conducted using SPSS 26.0 software and R language (version 4.5.1). Qualitative data were expressed as the number of cases (%), while quantitative data were presented as the mean ± standard deviation (x ± s). The *χ*^2^ test was used to compare the four groups of qualitative data, and the analysis of variance (ANOVA) was used to compare the four groups of quantitative data. A multivariate logistic regression analysis was used to investigate the relationship between endometrial VFI values and pregnancy outcomes; restricted cubic spline (RCS) curves were used to analyze the dose–response relationship between endometrial VFI values and pregnancy outcomes and to predict the appropriate range of endometrial VFI values. A *p*-value of < 0.05 was considered statistically significant.

## 3 Results

### 3.1 Comparison of general data and pregnancy outcomes

Endometrial volumes of Groups 1 and 3 were significantly lower than those of Group 4; the PI values of the right uterine artery and the left uterine artery in Groups 2 and 3 were significantly higher than those in Group 4; the miscarriage rates of Groups 3 and 4 were significantly lower than those of Group 1 (*p* < 0.05); there were no statistically significant differences in other general conditions and pregnancy outcomes among the four groups (*p* > 0.05) (Table 1).

**Table 1:** Comparison of general conditions and pregnancy outcomes among the four groups of patients.

Category	Group 1(VFI ≤ 0.041)	Group 2 (0.041<VFI ≤ 0.165)	Group 3 (0.165<VFI ≤ 0.461)	Group 4 (VFI>0.461)	*p*
Number of cycles	85	85	84	84	
Women’s age (years)	32.71 ± 4.45	32.74 ± 4.20	32.67 ± 3.72	31.46 ± 3.65	0.113
Graft intima thickness (mm)	9.47 ± 2.21	9.79 ± 2.31	9.55 ± 2.13	10.11 ± 2.10	0.232
Uterine endometrial volume (cm^3^)	2.94 ± 1.83	3.12 ± 1.65	2.81 ± 1.56	3.80 ± 2.24	0.002
Right uterine artery PI value	2.34 ± 1.25	2.62 ± 1.13	2.61 ± 0.95	2.23 ± 0.83	0.034
Right uterine artery RI value	0.82 ± 0.41	0.95 ± 0.71	0.90 ± 0.34	0.79 ± 0.21	0.121
Left uterine artery PI value	2.38 ± 1.46	2.59 ± 0.89	2.68 ± 1.00	2.21 ± 0.75	0.020
Left uterine artery RI value	0.79 ± 0.40	0.87 ± 0.17	0.88 ± 0.26	0.80 ± 0.19	0.053
Single cleavage embryo transfer rate (%)	9 (10.6%)	4 (4.7%)	4 (4.8%)	1 (1.2%)	0.054
Double cleavage embryo transfer rate (%)	24 (28.2%)	29 (34.1%)	27 (32.1%)	34 (40.5%)	0.398
Single blastocyst transfer rate (%)	24 (28.2%)	18 (21.2%)	18 (21.4%)	20 (23.8%)	0.681
Double blastocyst transfer rate (%)	28 (32.9%)	34 (40.0%)	35 (41.7%)	29 (34.5%)	0.59
Quality embryo transfer rate (%)	45 (52.9%)	56 (65.9%)	49 (58.3%)	51 (60.7%)	0.384
HCG-positive rate (%)	54 (63.5%)	45 (52.9%)	49 (58.3%)	47 (56.0%)	0.554
Clinical pregnancy rate (%)	48 (56.5%)	42 (49.4%)	41 (48.8%)	38 (45.2%)	0.523
Early abortion rate (%)	14 (29.2%)	8 (19.0%)	3 (7.3%)	3 (7.9%)	0.016

### 3.2 Logistic regression analysis of the independent risk factors influencing miscarriage

The single-factor logistic regression analysis indicated that the age of the female and the VFI value of the endometrium had a significant impact on the miscarriage rate after embryo transfer (*p* < 0.05). After conducting a multi-factor logistic regression analysis on the above variables and correcting for relevant confounding factors, the corrected OR values of Groups 3 and 4 compared to Group 1 were 0.210 (95% CI 0.055–0.805) and 0.227 (95% CI 0.059–0.870), respectively. This suggests that the miscarriage rates of Groups 3 and 4 were significantly lower than those of Group 1 (*p* < 0.05) (Table 2).

**Table 2:** Results of a binary logistic regression analysis with abortion as the dependent variable.

Variable	OR	95%CI	*p*
VFI grouping (Group 2 compared with Group 1)	0.573	0.210–1.562	0.277
VFI grouping (Group 3 compared with Group 1)	0.210	0.055–0.805	0.023
VFI grouping (Group 4 compared with Group 1)	0.227	0.059–0.870	0.031
Woman age (years)	1.095	0.982–1.222	0.103

### 3.3 Analysis of the RCS curve of VFI values in the endometrium and the risk of miscarriage

**Figure 2:**
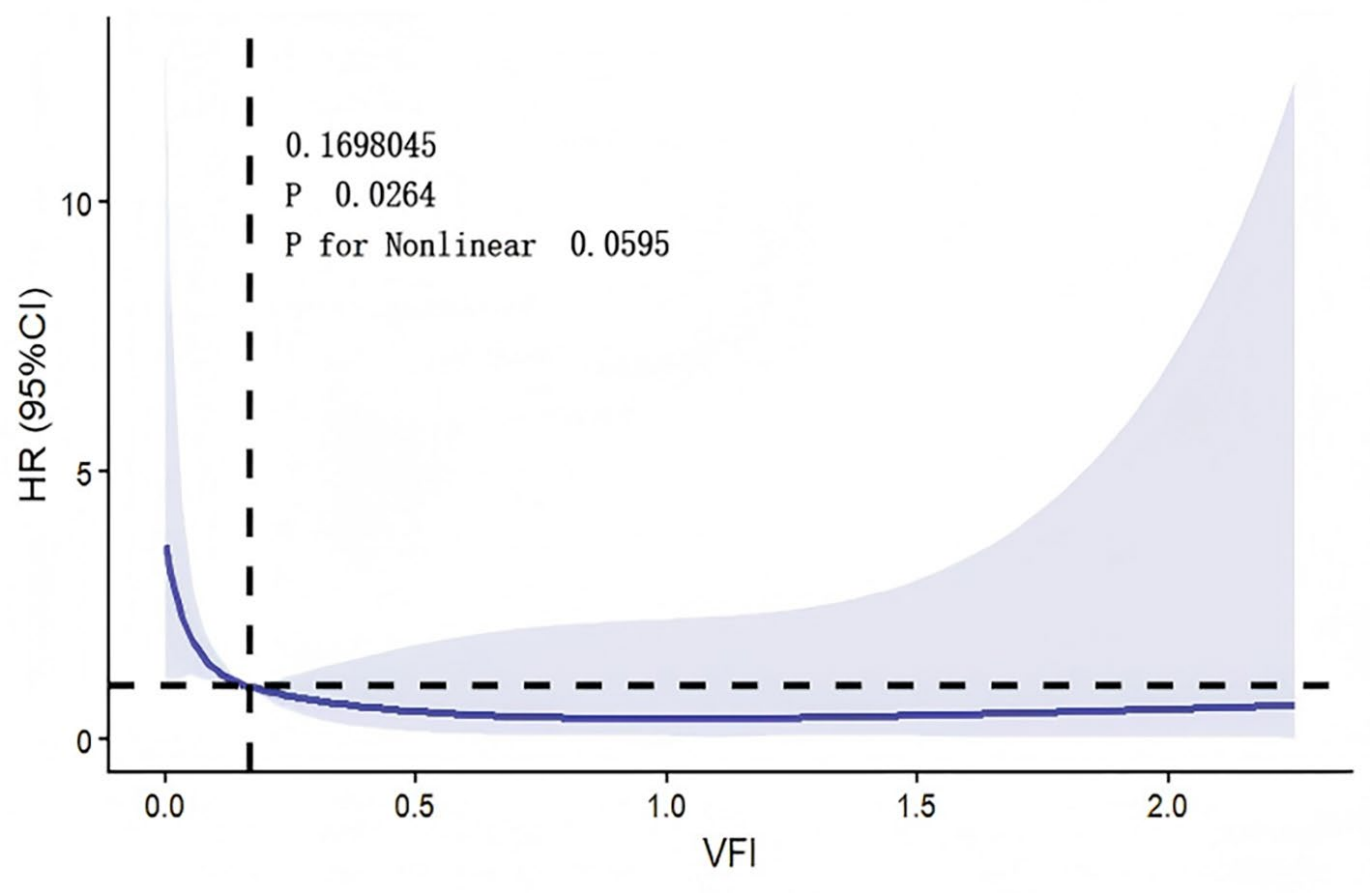
Abortion rate.

After correcting the endometrial volume to 2.74, the PI of the right uterine artery was 2.44, the RI of the right uterine artery was 0.86, the PI of the left uterine artery was 2.415, the miscarriage rate decreased, and the threshold was 0.17 (Figure 2).

## 4 Discussion

Endometrial receptivity is the key to successful embryo implantation and development (14–16). The current methods for evaluating endometrial receptivity include endometrial receptivity markers, such as endocytic protrusions; endometrial receptivity factors, such as levels of interleukins, epidermal growth factor, vascular endothelial growth factor, estrogen receptors, and progesterone receptors; and ultrasound (17). Ultrasound has the advantages of being rapid, economical, non-invasive, and capable of repeated dynamic examination, and is widely used in clinical diagnosis and treatment. Blood perfusion is an important factor affecting endometrial receptivity. Three-dimensional energy Doppler ultrasound blood flow imaging technology provides a reliable means for non-invasive quantitative assessment of endometrial blood perfusion. The endometrial VFI value can comprehensively reflect the degree of endometrial blood flow and its distribution characteristics. Compared with two-dimensional ultrasound and traditional color Doppler technology, endometrial VFI can more comprehensively reflect the blood perfusion status of the endometrium and can directly display the spatial relationship of the structure. This study found that the abortion rates of Groups 3 and 4 were significantly lower than those of Group 1 (*p* < 0.05). The multivariate logistic regression analysis revealed that, after correcting for confounding factors, the corrected OR values of Groups 3 and 4 compared to Group 1 were 0.210 (95% CI 0.055–0.805) and 0.227 (95% CI 0.059–0.870), respectively, suggesting that the abortion rates of Groups 3 and 4 were significantly lower than those of Group 1 (*p* < 0.05). This is consistent with the research results of Ng et al. (18). Vascular angiogenesis is active in the early stage of embryo implantation, and the expression of various angiogenesis-related factors increases, promoting endometrial decidualization and providing support for embryo development and the continuation of pregnancy (19). Vascular endothelial growth factor (VEGF) is a key cytokine for endometrial angiogenesis and repair, and it participates in angiogenesis through promoting endothelial cell proliferation and increasing vascular permeability (20). Its expression level is closely related to endometrial receptivity. Studies have found (21) that the endometrial VFI value is positively correlated with the expression of vascular endothelial growth factor. A decrease in endometrial VFI value indicates insufficient local blood perfusion, resulting in the downregulation of vascular endothelial growth factor expression, which, in turn, affects endometrial angiogenesis, endometrial receptivity, the transportation of nutrients and oxygen, and the development and maintenance of pregnancy after embryo implantation, thereby increasing the abortion rate.

By analyzing the dose–response relationship between the VFI value of the endometrium and the pregnancy outcome using the RCS analysis, it was found that, after correcting the endometrial volume to 2.74, the PI of the right uterine artery was 2.44, the RI of the right uterine artery was 0.86, the PI of the left uterine artery was 2.415, and the RI of the left uterine artery was 0.85, the VFI value of the endometrium was significantly linearly negatively correlated with the miscarriage rate (*p* < 0.05); when the uterine artery VFI value reached the threshold, as the VFI value of the endometrium increased, the miscarriage rate decreased, and the threshold was 0.17. The study by Wu et al. (22) found that the VFI of the endometrium was significantly increased in the pregnancy group, with the best predictive value being the endometrial sub-membrane VFI > 0.24, which was different from our best predictive value and might be related to the included research subjects, the timing of ultrasound detection, and the methods used.

In summary, the results of this study show that when the VFI value of the endometrium decreases, the miscarriage rate after frozen–thawed embryo transfer increases. When the VFI value of the endometrium is greater than 0.17, a better pregnancy outcome can be achieved. For infertile patients undergoing frozen–thawed embryo transfer with a hormone replacement cycle protocol, it is recommended to perform a three-dimensional energy Doppler ultrasound examination of the endometrial vascular blood flow index on the day before embryo transplantation, using a VFI of > 0.17 as an important reference indicator for evaluating endometrial receptivity and determining whether to proceed with embryo transfer. This study is a retrospective single-center study, and to avoid the influence of different endometrial preparation protocols on the results, only infertile patients who underwent endometrial preparation with a hormone replacement cycle protocol were included. There are certain selection biases and limitations. Future studies should increase the sample size and conduct multicenter, prospective randomized controlled trials comparing different endometrial preparation protocols to further validate the conclusions of this study and provide new insights into optimizing frozen–thawed embryo transfer strategies and improving the pregnancy outcomes of infertile patients.

## Data Availability

The original contributions presented in the study are included in the article/Supplementary material, further inquiries can be directed to the corresponding author.
